# Alcohol in Psoriasis—From Bench to Bedside

**DOI:** 10.3390/ijms22094987

**Published:** 2021-05-07

**Authors:** Zita Szentkereszty-Kovács, Krisztián Gáspár, Andrea Szegedi, Lajos Kemény, Dóra Kovács, Dániel Törőcsik

**Affiliations:** 1 Department of Dermatology, Faculty of Medicine, University of Debrecen, Nagyerdei krt. 98, 4032 Debrecen, Hungary; szkzyta@gmail.com (Z.S.-K.); gaspar.krisztian@med.unideb.hu (K.G.); aszegedi@med.unideb.hu (A.S.); kovacs.dora@med.unideb.hu (D.K.); 2 Division of Dermatological Allergology, Department of Dermatology, Faculty of Medicine, University of Debrecen, Nagyerdei krt. 98, 4032 Debrecen, Hungary; 3 HCEMM-USZ Skin Research Group, Department of Dermatology and Allergology, University of Szeged, Korányi fasor 6, 6720 Szeged, Hungary; kemeny.lajos@med.u-szeged.hu; 4 MTA-SZTE Dermatological Research Group, Eötvös Loránd Research Network (ELKH), Korányi fasor 6, 6720 Szeged, Hungary

**Keywords:** psoriasis, alcohol, addiction, compliance, genetic factors, keratinocytes, immune cells

## Abstract

Alcohol affects the symptoms, compliance and comorbidities as well as the safety and efficacy of treatments in psoriatic patients. In this review, we aim to summarize and link clinical observations with a molecular background, such as signaling pathways at the cellular level and genetic variations, and to provide an overview of how this knowledge could influence our treatment selection and patient management.

## 1. Introduction

Psoriasis, with a prevalence of 1–4%, is one of the most common chronic inflammatory skin diseases; however, it is not only the disorder of the skin but also a systemic one, associated with significant comorbidities that influence disease pattern, prognosis, treatment and management [1]. Importantly, the severity of psoriasis greatly affects the life quality of patients as well, where impairment is most commonly measured by the Dermatological Life Quality Index (DLQI) [2] and takes a prime position in patient management.

Regarding its clinical phenotypes, chronic plaque-type, guttate, erythrodermic and pustular psoriasis can be distinguished, while based on the localization, other variants such as inverse or flexural psoriasis, palmoplantar psoriasis, sebopsoriasis and nail psoriasis can be described as well [3]. According to the age of onset, psoriasis can be further subclassified into early-onset (≤40 years) and late-onset (>40 years) groups, underpinned by findings that a more pronounced genetic background is found in the early-onset one [4]. The severity of psoriasis may range from a few lesions to the involvement of the whole body and can be specified by several parameters, including the size of the affected area and inflammation of the lesions, which are all reflected in the Psoriasis Area Severity Index (PASI) score. Such scoring is the basis for distinguishing mild, moderate and severe psoriasis and has been widely used (in combination with the DLQI) for treatment selection and monitoring treatment response [5]. Therapeutic choices include topical therapies with classical alternatives such as keratolytics and local corticosteroids possibly in combination with vitamin D3 analogs [6] and calcineurin inhibitors. Further options cover phototherapy, oral systemic agents (methotrexate, acitretin, cyclosporine and small molecules, e.g., fumaric acid ester) and biological agents [7]. However, the International Psoriasis Council published a simplified statement in 2020, which instead of the mild, moderate and severe classification defines patients suitable for systemic therapy based on meeting at least one of three criteria: more than 10% of body surface area is affected, psoriasis at special sites (scalp, face, palms and soles or genitalia) and non-response to topical therapy [8].

In the development of psoriasis, an interplay between multiple genetic and environmental factors (e.g., stress, nutritional status and mechanical trauma) are involved, of which—despite an increasing knowledge—many are yet to be characterized in more details. The relationship between alcohol use and psoriasis is one of the prime examples for this, raising provoking questions such as, “Does psoriasis drive people into uncontrolled alcohol consumption?” or vice versa, “Does the presence of alcohol and alcohol-induced organ changes drive the development of psoriasis?”. Therefore, in this review, we aim to reveal the many situations in which alcohol consumption has to be taken into consideration for the successful management of chronic plaque-type psoriasis (later referred simply as psoriasis) patients. Moreover, we deliver a comprehensive overview of how the interaction of alcohol with symptom severity and medication efficacy may be determined at the level of molecular mechanisms and genetic background.

Using PubMed and Google Scholar with the term “psoriasis”—in combination with “alcohol”, “genetics”, “comorbidities”, “mental disorder”, “stress”, “adherence”, “compliance”, “keratinocyte”, “immune cell”, “management” and “therapy”—we focused on all relevant publications in English from the past 10 years (March 2011 to March 2021), included highly-cited older publications as well as additional reports from the references in systematic reviews.

## 2. Role of Alcohol in Psoriasis

It has been reviewed and reported in many studies that significant alcohol intake is an independent risk factor in the onset of psoriasis [9], presuming and partly revealing numerous mechanisms in the background of the disease. Alcohol use is also able to worsen the already-existing disease via increased tumor necrosis factor-α (TNFα) production and triggers alcohol-related disorders (e.g., alcoholic liver disease); furthermore, the treatment response in psoriasis is less favorable in cases of considerable alcohol consumption as these data have also been explored in a meta-analysis [10]. In alcoholics, the activity of pro-inflammatory cytokines was found to be elevated, which might contribute to the inflammatory process in psoriatic patients [11].

### 2.1. Alcohol and Its Relation to Psoriasis Symptoms at the Cellular Level

Ingested alcohol (i.e., ethanol) can be metabolized primarily to acetaldehyde by the alcohol dehydrogenase (ADH) enzyme family, catalase and cytochrome P450 isoform 2E1 (CYP2E1) and further oxidized into acetate and acetyl coenzyme A (acetyl-CoA) by the enzyme aldehyde dehydrogenase (ALDH) [12]. Acetyl-CoA serves as a precursor for the generation of acetone, which is formed by the decarboxylation of acetoacetic acid [13]. During ethanol metabolism, the formation of reduced nicotinamide adenine dinucleotide (NADH) is increased which delays the degradation of acetyl-CoA and thus elevates acetone blood levels [13]. Ethanol and its metabolites (acetaldehyde and acetone) can be both initiating and exacerbating factors at the cellular level in the inflammatory processes of psoriasis.

#### 2.1.1. Alcohol and Keratinocytes

It is known that from consumed and digested ethanol a measurable amount finds its way to and within the human skin, being secreted by the eccrine glands (primarily sweat glands) or during passive diffusion [14,15,16]. The potential effects of transdermal alcohol have been investigated in vitro and found that ethanol and its metabolite acetone increase the proliferation and mRNA expression of proliferation-associated genes (α5 integrin, cyclin D1 and keratinocyte growth factor receptor [KGFR]) in HaCaT keratinocytes and may thus increase the permeability of the skin, disrupting its barrier function [17]. In addition, alcohol also has an effect on lipid metabolism: the risk of high triglycerides is increased with increasing alcohol consumption, and thus it may also affect the lipid composition of the skin barrier [18].

Reactive oxygen species (ROS) generated in the skin of alcohol consumers during ethanol metabolism and acetaldehyde formation are able to regulate the mitogen-activated protein kinase/activator protein 1 (MAPK/AP1), the nuclear factor kappa-B (NF-κB) and the Janus kinase/signal transducers and activators of transcription (JAK/STAT) signal transduction cascades [19]. This oxidative stress, together with TNFα, one of the major cytokines in psoriasis, results in a positive feedback loop, leading to additional ROS formation and interleukin (IL)-1, IL-6 and IL-8 inflammatory cytokine production in primary human keratinocytes, thus contributing to the pathogenesis of psoriasis [20].

AP1 proteins are expressed in a differentiation-stage-dependent manner in keratinocytes. Acetaldehyde activates c-Jun mRNA and protein levels and induces Jun/AP1 DNA binding activity in oral keratinocytes, therefore, it is reasonable to suppose that ethanol may disturb keratinocyte proliferation and is involved in the aggravation of psoriasis [21,22].

The family of protein kinase C (PKC) isoenzymes regulates important cellular functions including cell growth, differentiation and cytokine production [23]. Characteristic PKC isoenzyme patterns were described in HaCaT cells during cell proliferation and differentiation [24] and it is generally accepted that the down-regulated PKC subspecies in psoriasis may be involved in aberrant cell growth and differentiation [25,26,27]. The interaction of ethanol with PKC is complex and the results are generally contradictory due to the various species/tissues and different alcohol exposures that were used in the experiments [28,29,30,31,32,33]; however, it is worth noting that through the regulation of PKCs, ethanol might negatively affect the cell cycle and tissue growth in psoriasis.

#### 2.1.2. Alcohol and the Immune System

Ethanol and its previously listed metabolites also affect both innate and adaptive immunity and may have a direct or indirect impact on immune cells.

Ethanol was found to augment the uric acid-induced NLR family pyrin domain-containing 3 (NLRP3) inflammasome activation and IL-1β production through the repression of the aryl hydrocarbon receptor (AhR) in macrophages [34], increase the proliferation and induce the release of interferon-γ (IFNγ) in lymphocytes and elevate the production of TNFα from peripheral blood monocytes and macrophages [15,19]. The soluble form of TNFα is released by the TNFα-converting enzyme (TACE), the overexpression of which was described in psoriatic lesions [35]. Furthermore, excessive alcohol consumption may contribute to its increased expression in peripheral blood mononuclear cells (PBMCs) and, in consequence, to elevated plasma soluble TNF- receptor 1 (sTNF-R1) concentrations in patients with psoriasis [36].

The relationship between keratinocytes and immune cells is well known in the pathomechanism of psoriasis. When supernatants of keratinocytes isolated from the skin of psoriatic patients were co-cultured with HUT 78 lymphocytes, findings showed increased protein levels of IFNγ, transformed growth factor-α (TGFα) and IL-6 after ethanol treatment, and suggested that ethanol also affects the epidermal and immune cell communication by triggering the T-helper (Th) 1 response [37].

Murine models also support a role for ethanol in skin inflammation. In vitro studies showed that the expression of C-C motif chemokine ligand 20 (CCL20) (an essential chemoattractant molecule in psoriasis) was induced in normal murine epidermal keratinocytes when the medium was supplemented with ethanol [38]. Moreover, the skin of ethanol-administrated hairless mice is characterized by attenuated skin hydration with significantly increased transepidermal water loss, up-regulated expression of TNF receptor 2 (TNFR2), decreased production of ceramide and type I collagen and increased plasma TNFα concentrations. In addition, anti-TNFα antibody treatment ameliorated the impaired skin barrier function in these mice [39]. Chronic ethanol feeding negatively affects both the number and the function of murine epidermal and dermal T cell subsets [40]. Immunostaining analysis revealed that in the skin of C57BL/6 mice receiving 5% ethanol in their drinking water, epidermal T-lymphocyte infiltration and IL-17A, TNFα and CCL20 mRNA expressions were increased. Moreover, when these mice received both ethanol and topical imiquimod (imiquimod-induced skin inflammation is currently the most widely accepted psoriasis animal model) [41], prominent epidermal thickening and more severe skin inflammation were observed with elevated expression of IL-17A, IL-22, IL-23 and S100A9 [38].

**Figure 1:**
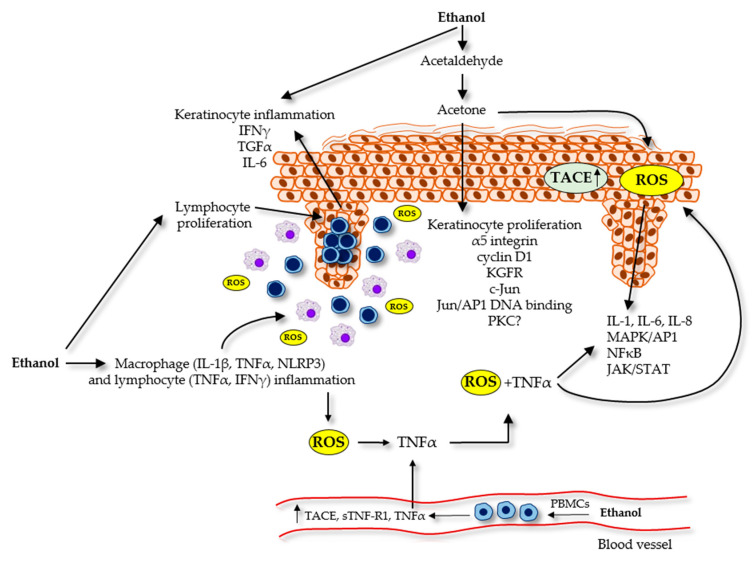
Overview of the potential role of ethanol and its metabolites at the cellular level in the pathomechanism of psoriasis. Ethanol and acetone increase the proliferation and mRNA expression of proliferation-associated genes in keratinocytes and disrupt skin barrier function. Ethanol and ROS formed during ethanol metabolism generate an inflammatory environment and trigger psoriasis by regulating different signal transduction pathways and inducing the production of various pro-inflammatory cytokines in lymphocytes, macrophages and keratinocytes. Excessive alcohol consumption may contribute to increased TACE expression in PBMCs and, in consequence, to elevated plasma sTNF-R1 and TNFα concentrations in patients with psoriasis.

Altogether, these data suggest that alcohol consumption can adversely affect psoriasis symptoms at the cellular level by influencing the proliferation, differentiation and skin barrier function of keratinocytes and inducing immune dysfunction via altering the production of inflammatory cytokines by immune cells. The potential role of ethanol and its metabolites in the pathomechanism of psoriasis at the cellular level is overviewed in Figure 1.

### 2.2. Alcohol and Patient Compliance

Patient compliance and therapeutic adherence are key factors in the comprehensive management of psoriasis and the definition of treatment success and disease prognosis. Patient adherence is defined as the consistent behavior of an individual that matches the recommendations agreed upon with the treating physician. Factors influencing treatment adherence can be patient-related, physician-related or external ones [42]. Many external factors may interfere with patient compliance, such as information provided to an individual on the disease, environmental influence or limitation caused by repetitive alcohol consumption (abuse), etc.; however, practical reviews examining psoriatic treatment adherence with an established scientific approach are limited.

In their open prospective study, Zaghloul and Goodfield assessed treatment compliance in psoriatic patients by direct questioning. They reported mean medication adherence to be around 60% and identified moderate alcohol intake (besides other factors such as being fed-up or forgetful, having facial symptoms and more extended disease form) as the main reason for non-adherence [43].

Carrol et al. suggested that in psoriasis the more an individual adheres to treatment, the better the reduction in disease severity [44]. While they examined the effect of therapeutic adherence to changes in psoriasis severity, they learned and concluded that non-adherence altered clinical trial data toward ineffectiveness, which might have been improved by special interventions such as motivations for self-reports or electronic monitoring [44].

In their critical review, Thorneloe et al. searched for the factors associated with and the extent of non-adherence in psoriasis. Keeping in mind the methodological limitations arising from the included studies, they drew a tentative conclusion that suboptimal treatment adherence was determined by psychological factors (e.g., psychological distress) rather than by quality of life alterations [45].

## 3. Role of Psoriasis in Alcohol Consumption

Evidence also exists that severe forms of psoriasis may lead to increased alcohol use [46]. In a robust search covering almost 40 years of reports, alcohol, along with other behavioral factors, was found to have an independent association with psoriasis that may influence several comorbidities of the disease as well [47].

### 3.1. Stress and Mental Disorders in Psoriatic Patients

Psychosocial stress, as one of the most important extrinsic factors that affect psoriasis, can prolong or even trigger psoriasis in susceptible individuals [3]. The etiopathogenesis of the psoriasis–psychological stress interaction includes the central and the peripheral hypothalamic–pituitary–adrenal (HPA) axis escalating inflammatory cytokines; activation of the sympathetic nervous system resulting in a defective adrenergic response and redistribution of leucocytes, moreover the stimulation of neuronal growth and alteration in neuropeptide and neurotrophin expression [48].

Chronic stress, however, exacerbates not just psoriasis itself, but also many mental disorders. The prevalence of psychiatric conditions in psoriasis may range from 24% to 90% [49]. A population-based cohort study in the UK found that patients with psoriasis have an increased risk of depression, anxiety and suicidality compared to the general population [50]. Out of these risks, the presence of depression escalates when pruritus is present [51], but in overviewing the relevant literature, data also show that more than 50% of the psoriatic population have sleep disorders [52] and up to 71.3% face sexual dysfunction [53]. Moreover, somatoform disorders, schizophrenia/other psychoses and bipolar disorder are also on the list of mental disorders linked to psoriasis [54]. It is needless to say that as a result of stress, quality of life diminishes and the psychosocial comorbidities promote more stress, a vicious circle that often precipitates substance dependence or abuse. However, despite the strong connection between alcohol consumption and psychological distress (associated with worry), only a small proportion of patients seek help or attend appointments for these matters [55].

### 3.2. Alcohol Consumption and Addiction in Psoriatic Patients

Susceptibility to different addictions seems to be increased in the psoriatic population. Conducting a study based on a self-reported screening test by a validated questionnaire of the six most common addictions in Germany with 102 psoriatic patients, Eyerich et al. found that the risk of alcohol abuse, nicotine abuse and gambling were significantly higher in the study group compared to the general population [56], defining the need for public health strategies and interdisciplinary approaches to tackle the vulnerability of psoriasis patients.

McAleer et al. investigated the association between alcohol intake and disease severity by applying validated questionnaires (Michigan Alcohol Screening Test, Alcohol Use Disorders Identification Test—AUDIT, CAGE) and biomarkers, such as γglutamyl-transferase (γGT) and carbohydrate-deficient transferrin (CDT), on a study cohort of 135 patients with moderate to severe chronic plaque psoriasis [57]. However, they did not find a significant correlation between excessive alcohol consumption and disease severity—in contrast to a study by Kirby et al. [58]—and revealed a nearly identical range (22–32%) of alcohol misuse among psoriatic patients [57]. Meanwhile, in another small study population using whole-blood phosphatidylethanol (PEth) to verify alcohol intake, the level of alcohol consumption correlated with the extent of psoriasis [59].

Applying the AUDIT and CAGE questionnaires [60] to facilitate the recognition of potential alcohol problems, we involved 214 psoriasis patients in our study to assess alcohol use in our psoriasis population; however, only 27 confirmed regular alcohol use, while 34 denied drinking alcohol at any time. As the prevalence of alcohol consumption was found to be over 40% in the Hungarian population [61], it is very unlikely that the results from our psoriasis population were indeed valid, further suggesting that integrating and dealing with the drinking habits of psoriasis patients faces great limitations.

## 4. Genetic Factors behind Alcohol Consumption in Psoriatic Patients

Explicit genetic background is an integral part of the etiology of alcoholism. The genetic determination based on twin studies is about 45–60% [62], and further data from adoption and animal studies also supports its importance [63,64,65].

In the pre-GWAS era, gene identification efforts faced substantial difficulties due to relatively small sample sizes and the low hit rate of the assumed candidate genes in association with alcohol use disorder (AUD) [66]; however, the introduction of GWAS arm-in-arm with the establishment of large biobanks and international collaborations brought a breakthrough in this field of research. According to the literature, there are two main categories of candidate genes selected for genetic association studies related to AUD: genes involved in the first category play a functional role in the central nervous system’s (CNS) response to alcohol or other addictive substances (gamma-aminobutyric acid type A receptor subunit gamma1 [GABRG1] and GABR subunit alpha2 [GABRA2], μ-opioid receptor 1 [OPRM1], etc.) [67,68,69]; while genes belonging to the second category are essential in alcohol metabolism (ADH4, ADH1B, ALDH2) [70,71,72,73].

**Figure 2:**
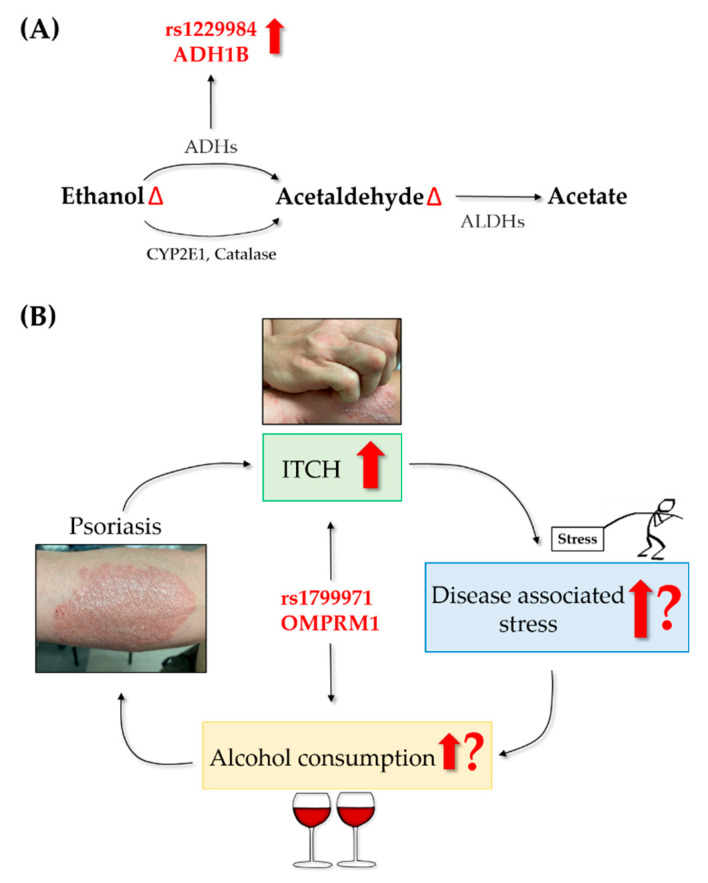
(**A**) Genetic variant rs1229984 (ADH1B gene), showing a significantly higher prevalence in our Hungarian psoriasis group (*n* = 776) compared to the Hungarian general population (*n* = 2967,leads to increased catalytic activity of the ADH enzyme that may alter the levels of ethanol and acetaldehyde. (**B**) Possible involvement of genetic variant rs1799971 (OPRM1 gene), found to increase the risk of familial psoriasis by twofold compared to sporadic cases in our Hungarian psoriasis group, in the psoriasis–itch–stress–alcohol consumption cycle.

While the genetic aspect is pivotal in the etiology of psoriasis, the question of whether the association between psoriasis and increased alcohol consumption may be driven by genetic factors has not yet been answered. Therefore, we conducted a population-based case-control study including 3743 (776 psoriasis and 2967control) subjects and evaluated the relationship between 23 increased alcohol intake and dependence-related single nucleotide polymorphism (SNP) in a Hungarian psoriasis group. Our results suggested that genetically defined high-risk individuals for alcohol consumption are more common in the psoriasis population [74]. We found that the frequency of C allele of the genetic variant rs1229984 in the gene coding ADH1B had a significantly higher prevalence compared to the Hungarian general population. This coding variant in ADH1B (Arg48His) results in a higher catalytic activity of the enzyme that may also lead to changes in the levels of harmful acetaldehyde following alcohol ingestion (Figure 2A). As supported by an Australian study involving 4597 twins, such carriers reported a lower prevalence of flushing after alcohol, consumed alcohol on more occasions, had a higher maximum number of alcoholic drinks in a single day and higher overall alcohol consumption than those with the less common A-allele (48His) [75].

By stratifying the psoriasis group, significant results were also found in the case of rs1799971 (OPRM1) when familial cases were compared to sporadic cases. Allele 118G increased the risk of familial psoriasis by twofold compared to sporadic cases. OPRM1 encodes the μ-opioid receptor, which upon activation by its ligands—such as opioids—modulates the dopamine system [76]. It is implicated in complex behavior patterns such as alcohol dependence [77,78] and alcohol dependence associated impulsivity [68]. In individuals carrying 118G, stimulation, sedation and positive mood levels after alcohol intake were significantly higher than in controls [79]. Importantly, OPRM1 is also involved in itch [80], a sensation reported by 70–90% of patients with psoriasis [81], calling for further studies to assess the psoriasis–itch–disease associated stress–alcohol consumption circle both at the level of clinical as well as of genetic studies [82] (Figure 2B).

## 5. Alcohol Affecting Psoriasis Therapies

While cyclosporine has no known interaction with alcohol and targeted therapies are a safe choice not only to achieve a specific and preferred therapeutic response but also in patients with impaired liver functions—the very rare cases of autoimmune hepatitis observed in patients receiving TNFα inhibitor treatments is not related to alcohol consumption [83]—prescribing acitretin and methotrexate needs special attention in psoriasis patients with alcohol problems.

### 5.1. Acitretin

Regarding acitretin, there is an enzymatic overlap between ethanol detoxification and retinoic acid biosynthesis. Although ADHs and ALDHs have different substrate preferences, the competition of ethanol, acetaldehyde and retinoids for the same metabolic enzymes potentially may result in teratogenicity and altered therapeutic response in psoriatic patients [84,85,86]. The formation of retinaldehyde from retinol also requires ADHs and retinol dehydrogenases (RDHs) and the oxidation of retinaldehyde to retinoic acid is catalyzed by retinaldehyde dehydrogenases (RALDHs or ALDHs) [87]. In therapy, acitretin has replaced its ethyl ester, etretinate, in the treatment of various keratinoid disorders such as psoriasis, as it is not stored in the adipose tissue, has a shorter half-life and is consequently eliminated more rapidly from the human body [88,89,90]. Interestingly, although etretinate is a prodrug of acitretin, Chou et al. showed that the conversion of etretinate to acitretin is not an irreversible process [91]. Moreover, in rats, etretinate formation from acitretin was significantly enhanced by simultaneous administration of varying amounts of ethanol, and plasma etretinate concentrations were correlated with ethanol doses but not with plasma acitretin levels [91]. Similar results were observed in in-vitro experiments on human liver cells [91] and in psoriatic patients receiving acitretin therapy, leading to the conclusion that alcohol consumption may enhance the etretinate formation from acitretin, in which ethanol could be an important contributing factor [92,93]. A possible underlying mechanism is that acetyl-CoA formed during ethanol metabolism enhances the conversion of acitretin to etretinate; thus, ethanol seems to act as an acetyl donor rather than an enzyme inducer during the ethyl esterification of acitretin [92,94].

### 5.2. Methotrexate

Methotrexate is known for causing non-alcoholic fatty liver disease (NAFLD); moreover, obesity (another risk factor for NAFLD) is also more prevalent among psoriasis patients [95,96], suggesting that indeed there may be a subgroup of patients predisposed to developing hepatotoxicity as a systemic manifestation of psoriasis itself and to methotrexate therapy. Although the regular follow-up of patients by measuring the levels of serum aminotransferases and performing regular FibroScan examinations makes methotrexate safe [97], elevated liver enzymes due to obesity and/or (possibly unreported) alcohol use may initiate the doctor to terminate the otherwise effective methotrexate treatment, even though studies suggest that moderate drinkers are not at risk when taking methotrexate. Among numerous studies with a relatively low number of patients often delivering conflicting results, a study of 11839 patients revealed that in patients taking methotrexate in a maximum dose of 25mg/week, consuming less than 14 units of alcohol did not appear to be associated with an increased risk, between 15 and 21 units was associated with a possibility of increased risk and drinking >21 units per week led to significantly increased rates of hepatotoxicity (adjusted HR [95% CI] 1.85 [1.17–2.93]) marked by elevated serum aminotransferases. For reference, a small glass of wine contains about 1.5 units, whereas a can of beer or cider is 2 units [98]. However, just as patients and their comorbidities differ, national and international guidelines also lack uniformity in advice on alcohol use with methotrexate [99,100].

## 6. Patient Management

Although DLQI covers the most important aspects of a patient’s life, questions that would reveal one’s relation to the use of obsessive agents and behaviors (such as “Do you feel stress, and if so, how do you find relief from it?”) are not included in it. Given that patients often feel themself humiliated when confessing about their alcohol-consuming habits, both physicians and researchers face great challenges when investigating this intimate topic; therefore, the general attitude that not speaking about it means it is not existing has to be changed. Indeed, although moderate alcohol consumption is a globally accepted social habit—except for in certain religious communities—the fact that alcohol itself is a toxic and psychoactive substance should also not be ignored.

While, except for methotrexate, regular alcohol use is not affecting the decision when selecting therapy for our psoriasis patients, increasing data confirms its possible impact on psoriasis symptoms, patient compliance and treatment effectiveness, for which we must address all potentially relevant barriers and provide thorough and supportive education for them. In their comprehensive guideline, Elmets et al. suggested that psoriatic patients should be counseled about the limitation of alcohol use that bears the risk for more severe disease and associated comorbidities (the strength of the recommendation was B and the level of evidence was II–III). Furthermore, they concluded that since the amount of alcohol also has an impact on concomitant pharmaceutical medications, it should therefore also be considered (the strength of the recommendation was B and the level of evidence was II–III). They further recommended that those with alcohol and concomitant nicotine dependency should be referred to healthcare specialists for further support (the strength of the recommendation was A). Additionally, they described that since the avoidance of alcohol consumption can lead to the improvement of psoriasis, the dermatologist should possibly play a supportive/helping role in informing the patients of that matter [47].

The need to offer help for psoriatic patients is further emphasized by Svantröm et al., who by introducing a neuro-endocrine approach in the alcohol dependence of psoriatic patients, pointed out that several therapeutic modulators (e.g., dopamine [DA] 2 receptor [R] and serotonin 5-hydroxytryptamine 2R [5-HT2R] antagonists and selective serotonin reuptake inhibitors [SSRIs]) used in the treatment of alcoholism may affect the neuronal transmission networks and biochemical pathways of alcohol metabolism and modulate the number of biogenic amine receptors and circulating pro-inflammatory cytokines, with a possible effect also on the inflammatory process in psoriasis. As so, they proposed the idea that targeting neurotransmitter networks involved in both alcohol intake and the inflammatory process could not only treat addiction but serve as a potential (adjuvant) psoriasis therapy as well [101].

## 7. Conclusions

In summary, the contribution of alcohol to the development of psoriasis is unquestionable on every level (genetic and cellular level, disease onset and severity, treatment options and adherence, prognosis, etc.). While the very limited “benchside” data suggests further potential in addressing mechanisms with both patho(physiological) and therapeutic relevance, from the clinician’s point of view, multidisciplinary strategies are needed to identify and manage the affected population all the way from the “barside” to the “bedside” and beyond. We strongly believe that supporting patients to make lifestyle changes may lead to rewarding therapeutic accomplishments.

## Abbreviations

5-HT2R	5-hydroxytryptamine receptor 2A
Acetyl-CoA	Acetyl coenzim A
ADH	Alcohol dehydrogenase
AhR	Aryl hydrocarbon receptor
ALDH	Aldehyde dehydrogenase
AP1	Activator protein 1
AUD	Alcohol use disorder
AUDIT	Alcohol use disorders identification test
CAGE	Cutting down, Annoyance by criticism, Guilty feeling, and Eye-openers
CCL20	C-C Motif chemokine ligand 20
CDT	Carbohydrate-deficient transferrin
c-Jun	Proto-oncogene c-Jun
CNS	Central nervous system
CYP2E1 DA	Cytochrome P450 isoform 2E1 Dopamine
DLQI	Dermatological life quality index
GABRA2	Gamma-aminobutyric acid type A receptor subunit alpha2
GABRG1	Gamma-aminobutyric acid type A receptor subunit gamma1
HPA-axis	Hypothalamic–pituitary–adrenal axis
IFNγ	Interferon-γ
IL	Interleukin
JAK	Janus kinase
KGFR	Keratinocyte growth factor receptor
MAPK	Mitogen-activated protein kinase
NAD	Nicotinamide adenine dinucleotide
NAFLD	Non-alcoholic fatty liver disease
NF-κB	Nuclear factor kappa-B
NLRP3	NLR family pyrin domain containing 3
OPRM1	μ-opioid receptor 1
PASI	Psoriasis Area Severity Index
PBMC	Peripheral blood mononuclear cell
Peth	Phosphatidylethanol
PKC	Protein kinase C
RALDH	Retinaldehyde dehydrogenase
RDH ROS	Retinol dehydrogenase Reactive oxygen species
SNP	Single nucleotide polymorphism
SSRI	Selective serotonin reuptake inhibitor
STAT	Signal transducers and activators of transcription
sTNF-R1	Soluble TNF-receptor 1
TACE	TNFα-converting enzyme
TGFα	Transforming growth factor-α
Th	T-helper
TNFR	Tumor necrosis factor receptor
TNFα	Tumor necrosis factor-α
γGT	γglutamyl-transferase
